# FRAP Analysis: Accounting for Bleaching during Image Capture

**DOI:** 10.1371/journal.pone.0042854

**Published:** 2012-08-09

**Authors:** Jun Wu, Nandini Shekhar, Pushkar P. Lele, Tanmay P. Lele

**Affiliations:** 1 Department of Chemical Engineering, University of Florida, Gainesville Florida, United States of America; 2 Department of Molecular and Cellular Biology, Harvard University, Cambridge, Massachusetts, United States of America; Baylor College of Medicine, United States of America

## Abstract

The analysis of Fluorescence Recovery After Photobleaching (FRAP) experiments involves mathematical modeling of the fluorescence recovery process. An important feature of FRAP experiments that tends to be ignored in the modeling is that there can be a significant loss of fluorescence due to bleaching during image capture. In this paper, we explicitly include the effects of bleaching during image capture in the model for the recovery process, instead of correcting for the effects of bleaching using reference measurements. Using experimental examples, we demonstrate the usefulness of such an approach in FRAP analysis.

## Introduction

The FRAP technique is a popular technique for investigating dynamics of protein diffusion and binding in living cells [1]–[8]. FRAP experiments involve bleaching of fluorescently labeled proteins in a pre-chosen location inside the cell with a high intensity laser pulse. When proteins are transiently bound to structures in the photobleached spot, the fluorescence recovers owing to exchange between fluorescently labeled diffusing molecules in the cytoplasm or membrane with the bound photobleached molecules in the bleached spot. The recovery curve can be fit to models to estimate transport and binding parameters. The accurate modeling of FRAP experiments and issues with parameter estimation are active areas of interest [2], [9]–[17].

The approach to fit FRAP experiments to mathematical models involves a suitable normalization of the experimental data [18]. For example, if 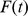 is the fluorescence in a spot in the cytoplasm, and bleaching occurs at 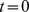, then one way to normalize the signal is 
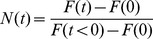
. Here, the denominator represents the amount of fluorescence that should theoretically recover after photobleaching assuming one waits long enough in the experiment (i.e. 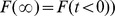, while the numerator represents fluorescence that has recovered at any time. The assumption can be made in most cases that the bleaching pulse at 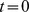 itself does not alter the total fluorescence significantly. If the experiment is then stopped at time 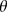
(when the fluorescence appears to visually plateau), in many cases it is found that 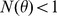 i.e. complete fluorescence recovery does not occur. If 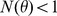, the usual procedure is to calculate the so-called immobile fraction 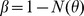; the hypothesis is that there is a sub-population of fluorescent molecules in the bleached spot that do not recover to any measurable extent over the time 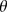. While this approach is widely followed in the literature and may be applicable for many situations, it is obvious that if there was significant bleaching as a result of the image capture process itself, then 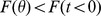 even though there is no real immobile fraction.

Of all the different experimental complications that make FRAP analysis difficult, the undesirable decay of the fluorescence due to the image capture process itself has received little attention. Typically, the decay is ‘corrected’ by dividing the observed signal by the overall signal in the cell. This procedure can potentially invalidate the fitting of mathematical models to FRAP data owing to the arbitrary correction of experimental data with another time-varying curve. If the effect of bleaching during image capture is significant and no correction to the data is applied, then this can invalidate the fitting because the mathematical models do not include the effect of photobleaching during image capture. Either way, neglecting the effect of photobleaching during image capture has the potential to render serious errors in the estimation of kinetic or transport parameters from the FRAP experiment. In this paper, we take the view that mathematical models for FRAP analysis should *explicitly* account for the effects of bleaching during image capture instead of relying on corrections to data, or on the perfect experiment that does not suffer from the effects of photobleaching. We develop models that should be generally applicable and provide an experimental demonstration on how to use the models. The analysis discussed here can help bring greater clarity into the interpretation of FRAP experiments.

## Materials and Methods

### Cell Culture, Plasmids and Transfection

NIH 3T3 fibroblasts were cultured in DMEM (Mediatech, Manassas, VA) with 10% donor bovine serum (Gibco, Grand Island, NY). For microscopy, cells were cultured on glass-bottomed dishes (WPI, Sarasota, FL) coated with 5 µg/ml fibronectin (BD Biocoat™, Franklin Lakes, NJ) at 4°C overnight. The EGFP-VASP plasmid was transiently transfected into NIH 3T3 fibroblasts with Lipofactamine™ 2000 transfection reagent (Life Technologies, Invitrogen, Carlsbad, CA).

### Confocal Microscopy and FRAP

Cells expressing EGFP-VASP were imaged on a Leica SP5 DM6000 confocal microscope equipped with a 63X oil immersion objective. A 488 nm Argon laser applied at 50% power was used to photobleach the focal adhesion in 5 iterations. Cells were maintained at 37°C in a temperature, CO_2_ and humidity controlled environmental chamber for all imaging experiments.

### FRAP Analysis

A program for fitting the models in this paper to data is available on request.

## Results

### Modeling Bleaching during Image Capture

We first consider the situation where fluorescence imaging is performed on a live cell. If an image is captured for an exposure time 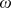, then the fluorescence concentration in the cell will decrease from an initial value of 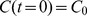 in this time according to the kinetic expression [19]

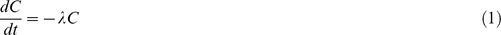
Where 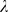 is the photobleaching rate constant (s^−1^). The precise value of 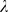 will depend on imaging conditions (i.e. laser power, magnification etc). At the end of the exposure time 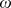, the concentration is 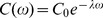. Consider an experiment involving imaging of the entire cell over
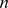
images with a time interval of 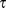 between images. The 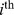 image capture is assumed to occur in the time interval 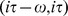. Then applying Eq. 1 for imaging at each time point, the formula for the concentration at the end of the 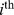
time interval is
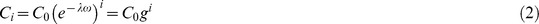
where 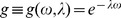.

**Figure 1:**
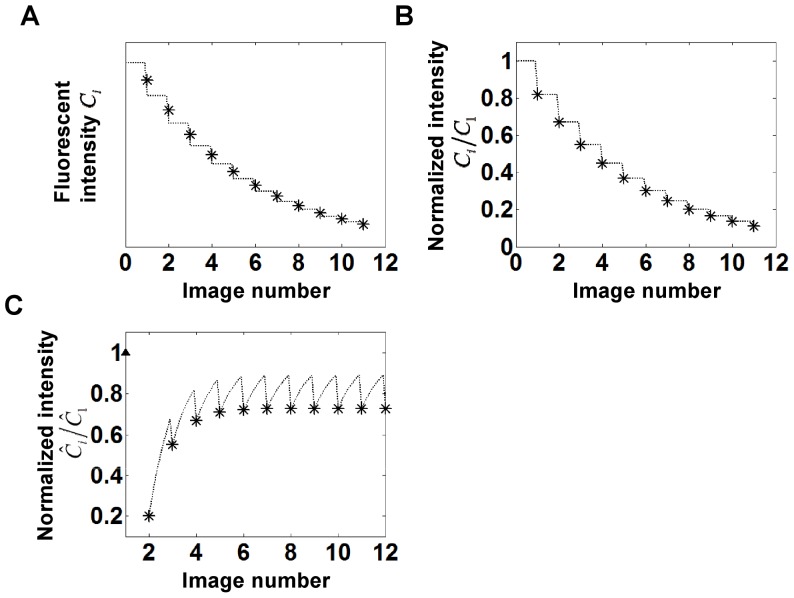
Effect of bleaching due to image capture on measured fluorescence. (A) shows calculations of Eq (2) for 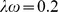 and 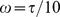 (as the actual concentration is not measured in experiments, the value of 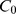 is not relevant). The dotted lines indicate the actual dynamics including the decay of the fluorescence due to bleaching during image capture. * indicates the averaged concentration in an image. B) Dotted curves are normalized concentrations calculated from Eq (3). Normalizing average concentrations with the concentration in the first image yields similar dynamics, except the effect of averaging on the measured concentration is cancelled (this is discussed more in the text) such that the normalized average fluorescence is equal to 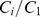. (C) Hypothetical effect of bleaching during image capture on FRAP recovery. The dotted curve is the actual dynamics consisting of (unobserved) recovery interspersed by bleaching during image capture, * indicates measured intensity. The solid triangle at 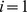 indicates the normalized initial intensity before photobleaching.



The time evolution of the concentration predicted by equation (2) for a hypothetical experiment is shown in Figure 1A. Because the imaging occurs over a time interval 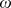, the *measured fluorescence* in the 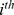 image is proportional not to 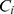 but rather to the average concentration over 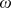 given by 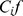, where 
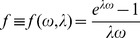
. However, as is common practice, the fluorescence in subsequent images is normalized to the fluorescence in the first image (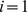) and so the factor 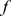 cancels, making the *normalized* fluorescence proportional to the ratio of concentrations 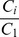. Figure 1B illustrates how normalization scales the hypothetical data from Figure 1A. Noting this requirement for normalization, the normalized fluorescence in a whole-cell imaging experiment obeys the equation

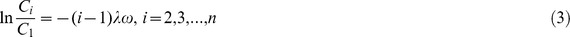
Eq. 3 allows the straightforward estimation of 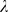 (assuming 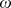 is known). Alternatively one could capture one image for a long enough 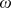 causing significant bleaching due to image capture; this suffers from potential heating artifacts though and may not be as reliable as the procedure suggested by Eq. 3.

### FRAP Model to Account for Photobleaching due to Image Capture

When the FRAP experiment involves selective photobleaching of bound molecules (such as molecules bound to a microtubule tip [20], or in a focal adhesion [21]–[23], or at a promoter array [7], [24]) the recovery occurs through diffusive transport of free protein molecules (in the cytoplasm, nucleoplasm or membrane) followed by exchange with bound molecules. A commonly encountered situation is where the exchange between bound and free protein is far slower than diffusive transport into the photobleached spot and the concentration of the free protein is unaffected by the exchange process owing to the large pool of free molecules compared to bound molecules [23], [25]. In this paper, we develop the modeling approach for this situation (the approach is generally applicable as discussed later).

We consider first the situation where bleaching during image capture is not significant. The equation describing the recovery process is (assuming that the free concentration is well-mixed and constant, and diffusion is very fast compared to binding)
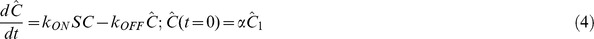
where 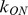 is the rate constant for binding, 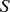 is the binding site concentration (which is assumed to be constant), 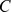 is the cytoplasmic (or membranous) diffusing concentration and 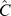 is the bound concentration in the photobleached spot. The initial condition reflects the fact that the photobleaching pulse reduces bound fluorescent molecules from an initial concentration of 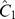 to 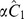 with 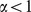 (the subscript 1 for the initial concentration anticipates the development in equation 6). The solution to this equation is



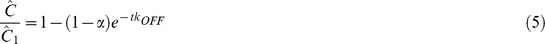
The typical approach in the literature is to normalize the experimental data as 
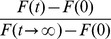
 and then fit it to 
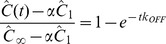
. The parameter estimated from the data is 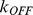.

However, if there is bleaching during the image capture process itself, then as illustrated in Figure 1C, the dotted curves are the actual dynamics consisting of (unobserved) recovery interspersed by bleaching during image capture leading to the measured recovery (indicated by (*)). It is necessary then to model the unobserved dynamics, consisting of recovery between time intervals of image capture and also the bleaching due to the image capture process itself to predict the observed recovery dynamics. Fitting such a model to the data has the advantage of faithfully capturing the recovery process, and eliminating the need for arbitrary corrections to the data (such as correcting the recovery signal by dividing with the total cell intensity which decays due to bleaching during image capture).

We now consider the time-evolution of the fluorescent bound concentration under the effects of bleaching due to image capture. Consider three images: one taken just before the photobleaching pulse corresponding to a final concentration of 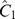 (‘final’ refers to the fluorescent bound molecule concentration at the end of the image capture), the second image immediately after the photobleaching pulse corresponding to a final concentration of 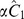 and the third image whose capture begins at a time interval of 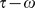 where 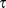 is the time interval between successive images (based on the rationale developed in modeling the whole-cell bleaching experiment). The fluorescent bound concentration just before the third image capture begins is 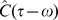. Because 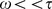 (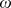
∼milliseconds and 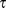
∼second), we can approximate 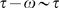 in Eq. 5 yielding 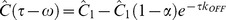. When imaging starts, photobleaching occurs due to image capture, and the concentration at 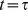 is 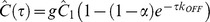; 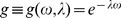 as before. Here we have made the assumption that the recovery itself occurs to a negligible extent in the time interval 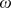 compared to fluorescence decay due to bleaching during image capture. This is reasonable considering that 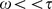, and the recovery time scale is much larger than 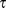.

The exchange process in the next time interval 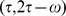 is still described by the differential equation in Eq. 5 but now with an initial condition 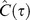. Extending this logic to the 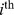 image, it is possible to calculate the concentration 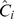 at the end of the 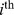 image capture as shown below (we note again that 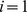 indicates the concentration at the end of image capture just before the photobleaching pulse, 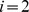 indicates the concentration at the end of image capture just after the photobleaching pulse):
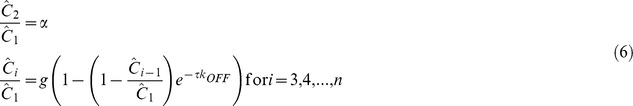



As before, the ratio of the measured fluorescence in the 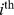 image to the fluorescence in the first image is proportional to (and should be fit to) the concentration ratio derived in Eq. 6.

An interesting point here is that the model predicts a steady state for the fluorescence recovery despite the fact that image capture results in periodic bleaching. Such a steady state will be reached when the fluorescence lost due to bleaching due to image capture is balanced by recovery in between images. This yields the equality (*N* represents any image collected in the steady state portion of the recovery curve) 
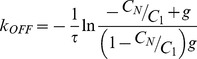
. At steady state, if the fluorescence intensity is known and the bleaching function 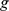 is determined from experiment, it is possible to calculate 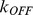 with this equation. This of course requires prior knowledge of the model that describes protein exchange in the spot (in this case, Eq. 4).

### FRAP Model to Account for Bleaching of Free Protein

Eq. 6 describes FRAP recovery when photobleaching during image capture is significant. In deriving these equations, we have assumed that the free protein concentration 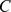 is unaltered by the bleaching. This assumption is typically valid if we consider that the bleaching during image capture occurs predominantly at the focal plane (where the laser beam is focused in a confocal microscope) and progressively less outside the bleached spot. As the space where the free molecules diffuse is well mixed on time scales of exchange with bound protein (this is the assumption underlying Eq. 4), it is reasonable to expect that the concentration of free molecules will decrease much less due to image capture than proteins present in a bound spot enclosed in the thickness of the focal plane (such as a focal adhesion or a receptor binding to a promoter array in the nucleus; this is discussed more in Supporting Information S1). The assumption that the free protein is not changing in concentration due to image capture can also be checked by measuring the fluorescence of free molecules as demonstrated in the experimental example later.

When the free molecules are also bleached during image capture, we let 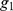 and 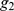 be the bleaching functions for bound and free proteins (corresponding to different ‘effective’ values of 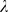: 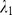 and 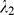; see Supporting Information S1). Then, the free concentration decreases similar to Eq. 3
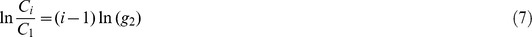



We continue to make the assumption that the free concentration is well-mixed, and unaffected by the exchange process itself with bound protein because of the large pool of free molecules compared to bound molecules. Using Eq. 7 with Eq. 4 for the unobserved concentration between successive images and accounting for bleaching, the bound concentration is
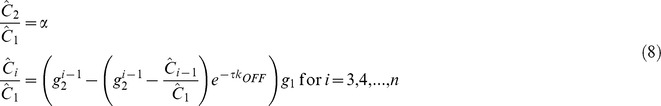



### FRAP Model to Account for an Immobile Fraction

As discussed above, assuming that the free protein pool is unaffected by the imaging process, the recovery should reach a steady state. This model, however, assumed that all of the molecules in the bleached spot were able to exchange with the cytoplasmic pool of molecules on a single time scale (∼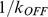). In many experimental situations, it is observed that the recovery is not complete, suggesting the presence of an ‘immobile’ fraction in the bleached spot. Then the full solution (including bleaching of the free pool) is
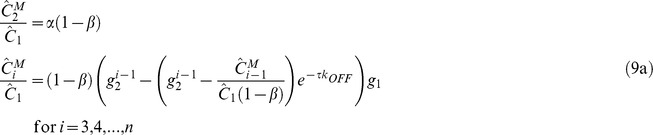


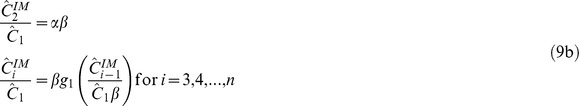



Here 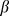 is the immobile fraction. 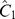 is the total concentration ( = 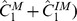, 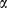 still represents the fraction of fluorescent bound molecules bleached. The contribution of mobile and immobile pools to the recovery need to be separately accounted for as shown in Eq. 9a and 9b (the superscript M refers to the mobile fraction, IM refers to the immobile fraction). The fluorescence intensity in a FRAP experiment normalized to the initial fluorescence just before the photobleaching pulse should be fit to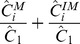.

### Calculations of Normalized Recovery: the Behavior of Eq. 6a and 6b

**Figure 2:**
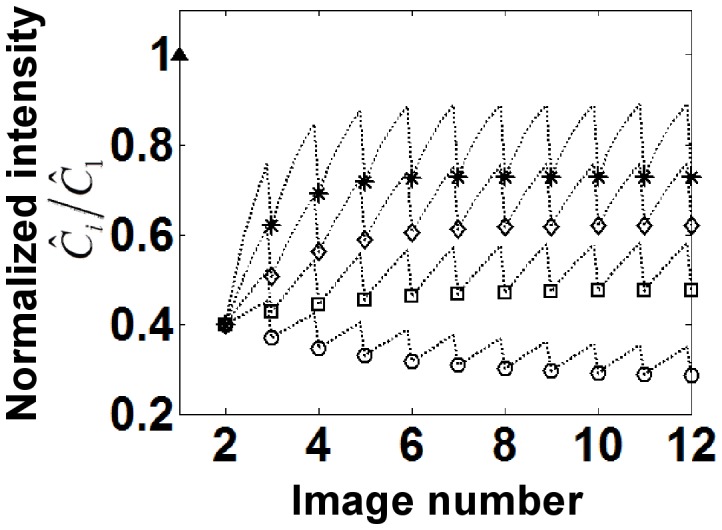
Solutions to Eq. 6 showing how bleaching during image capture can give the erroneous impression of an ‘immobile’ fraction. Recovery curves are shown with 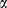 = 0.4, 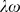 = 0.2, 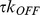  = 1 (*), 0.5 (◊), 0.25 (□) and 0.1 (○) (from top to bottom). For plotting purposes, 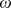 is assumed to be 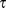/10. The solid triangle at 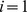 indicates the normalized initial intensity before photobleaching.

We explored the behavior of Eq. 6a and 6b numerically. As seen from Eq. 6, the recovery process depends on the parameter group 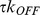, the parameter 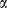, and the parameter group 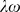. Fixing 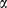 = 0.4 (a typical value for bleaching in experiments) and assuming 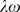 = 0.2, solutions to Eq. 6 are plotted for different values of 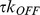 (Figure 2). Because 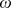 is kept constant ( = 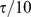), the value of 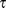 can be thought of as constant in Figure 2 (although its actual value or that of 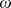 is not relevant since the solution does not depend on their individual values).

Figure 2 shows that when too many images are collected over the characteristic time scale of the recovery process (i.e. 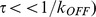, there is a significant decrease in net recovery owing to bleaching during image capture. In this situation, recovery due to protein exchange between successive image captures does not occur significantly owing to frequent photobleaching resulting in a steady state with low recovery. The extent of recovery increases with increasing 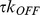. Thus, the photobleaching process during image capture itself can create an erroneous impression of an ‘immobile’ fraction.

### Effect of the Immobile Fraction on FRAP Recovery under Minimal Bleaching of Free Protein

**Figure 3:**
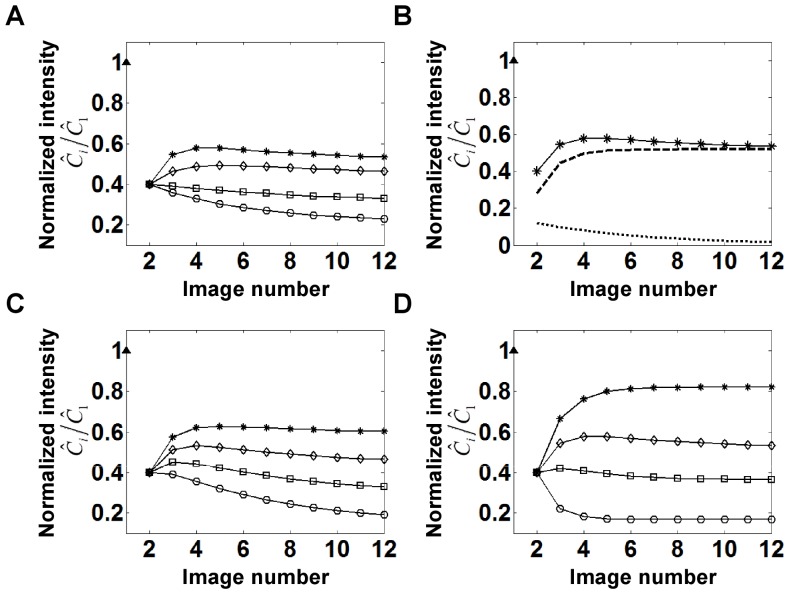
Solutions to Eq. 9a and 9b that account for the presence of an actual immobile fraction. (A) Observed recovery curves with 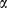 = 0.4, 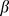 = 0.3 (immobile fraction), 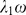 = 0.2, 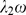 <<1 (i.e. negligible photobleaching of the cytoplasmic molecules such that 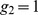) and 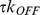  = 1 (*), 0.5 (**◊**), 0.25 (**□**), 0.1 (**○**) (from top to bottom). (B) Illustration of behavior of mobile (dashed curve) and immobile fractions (dotted curve) during recovery for 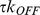 = 1 (* indicates total intensity). The immobile fraction can be seen to decay due to bleaching during image capture, resulting in a decrease in the total fluorescent intensity. (C) Effect of the immobile fraction on the observed recovery curves. 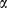 = 0.4, 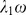  = 0.2, 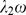 <<1, 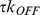  = 1 and 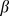 = 0.2 (*), 0.4 (**◊**), 0.6 (**□**), 0.8 (**○**) (from top to bottom). Pronounced transients are observed in the recovery. (D) Effect of the bleaching function 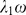 on recovery. Observed recovery curves with 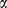 = 0.4, 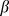 = 0.3, 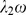 <<1, 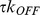 = 1, and 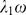 = 10^−6^ (*), 0.2 (**◊**), 0.46 (**□**), 1.1 (**○**) (from top to bottom). Solid triangles at 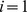 in all figures indicate the normalized initial intensity before the photobleaching.

Figure 3A shows calculations of recovery in the presence of an immobile fraction. The parameter values are identical to Figure 2, but solutions to Eq. 9a and 9b are plotted along with 30% immobile fraction i.e. 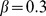. As seen, the recovery does not reach a steady state in comparable number of images (compare with Figure 2). Unlike the results in Figure 2, Figure 3A shows the presence of a peak in intensity such that the fluorescence intensity initially increases but then decreases. This is due to the fact that the immobile fraction (which by definition cannot exchange during the recovery process) continues to get bleached during the imaging process (as indicated by the decaying dotted curve in Figure 3B). Also, due to the bleaching of the immobile fraction, there can also be parameter conditions where the total intensity decays instead of recovering (see decaying curves in Figure 3A). For Eq. 9a and 9b, a steady state is achieved only when the immobile fraction is completely bleached.

Figure 3C shows the effect of the immobile fraction itself on the recovery curve. With an increasing immobile fraction, the recovery transients show a pronounced decay, and for high enough values, the recovery falls below the initial fluorescence value. Figure 3D shows the effect of 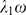 on the recovery process; the extent of photobleaching during image capture again significantly decreases the net recovery and a maximum in fluorescence intensity is predicted for some parameter values.

### Analysis of Focal Adhesion Protein Exchange

**Figure 4:**
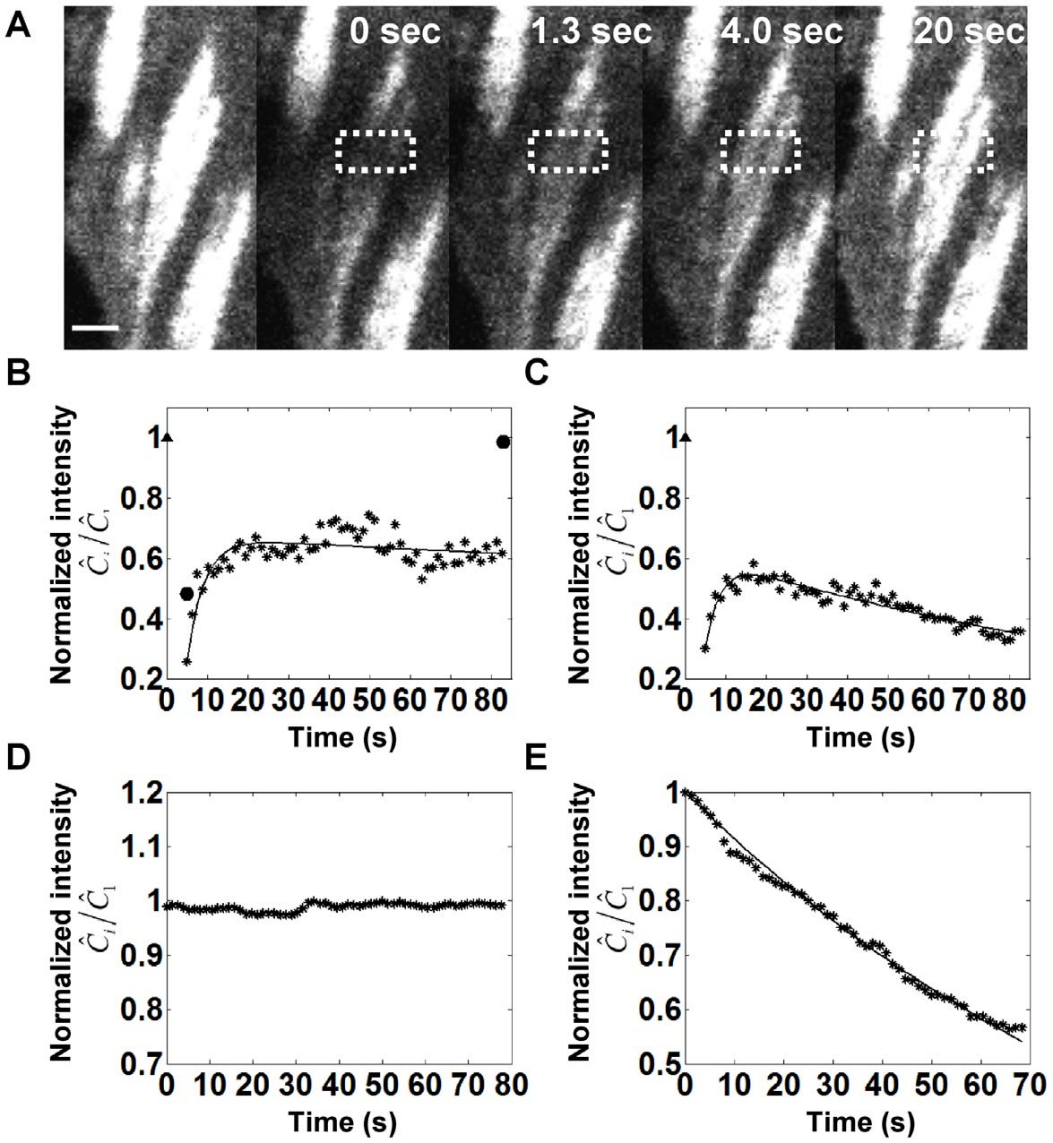
Example of the application of Eq. 9a and 9b for analyzing a GFP-VASP FRAP experiment. (A) Captured images from a FRAP experiment in an NIH 3T3 fibroblast expressing GFP-VASP. The box shows the bleach spot. Scale bar, 1 µm. (B) Observed recovery with only slight apparent bleaching of cytoplasmic free molecules (see D) due to image capture. The excitation laser power was 4%. The solid curve is the fitting of the data to the model in Eq 9. The immobile fraction 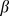 = 0.026 was estimated from a separate experiment (solid circles) in the same focal adhesion as described in the text. The value of 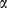 was determined from the fluorescence values before and immediately after the bleach, 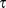 = 1.3 s. The fitting yielded the parameters 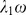  = 0.0902, 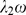 = 0.0014, 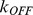 = 0.17 s^−1^. (C) Observed recovery in the same focal adhesion from a second FRAP experiment with apparent bleaching of the free molecules. The excitation laser intensity was increased to 10%. The fluorescence is observed to go through a peak and then decrease due to bleaching caused by image capture. The fitting of the data to the model gave the parameters 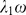 = 0.13, 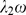 = 0.009, 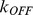 = 0.18 s^−1^. The value of 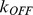 is very close to that estimated from the fitting in (B) thus validating the model. Solid triangles in (B) and (C) indicate the normalized initial intensity before the photobleaching. (D) Fluorescent intensity profile in the cytoplasm (free molecules) in experiment (B), which shows there is no detectable photobleaching of cytoplasmic molecules. (E) Fluorescent intensity profile of the cytoplasm (free molecules) in experiment (C) showing a clear decrease in the concentration due to pronounced bleaching. Fitting of the cytoplasmic intensity to Eq. 3 yields the bleaching parameter 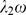  = 0.011, which is close to the value determined from the fitting in (C). The model for the cytoplasmic intensity was fit to 70 of the 80 seconds for which the data was collected (corresponding to 53 measurements); the first 10 seconds showed a significant deviation possibly due to deviations in focus.

As an example of the application of the model above, we performed FRAP analysis of the focal adhesion protein GFP-VASP. As we have shown before [23], the recovery curves in the case of focal adhesion proteins yield the parameter 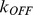. We first measured the immobile fraction in the chosen adhesion (Figure 4) by performing an initial bleach, capturing a single image immediately after bleach and a second image ∼80 seconds later (when the recovery transients were determined to reach a steady state). The immobile fraction was calculated from the formula 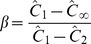 and was found to be 0.026 (solid circles in Figure 4B show the normalized concentrations immediately after bleach, 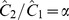, and after recovery, 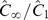).

Next, FRAP analysis was performed on the same focal adhesion in which 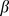 was measured above. The unknown parameters (as seen from Eq. 9) are 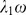, 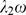 and 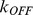. First, a FRAP experiment was performed such that relatively little photobleaching of the cytoplasmic molecules occurred during image capture. The intensity of the free protein was confirmed to be approximately constant (Figure 4D). Figure 4B shows that the model fit satisfactorily captures the recovery and the subsequent slight decline in the fluorescence recovery. The recovery is substantially less than the recovery observed in the experiment to calculate the immobile fraction above, suggesting an effect of bleaching due to image capture on the bound fluorescence in the focal adhesion. If this data was used to erroneously calculate the immobile fraction, it would yield a value of 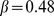, a significantly different value than the actual value determined above.

**Figure 5:**
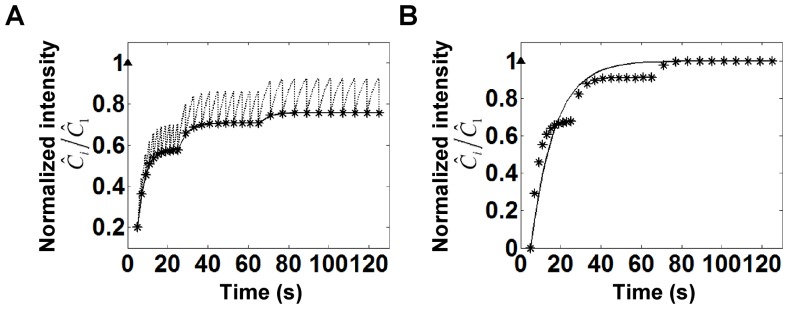
Predicted FRAP dynamics with changing time interval 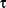. (A) Recovery curve calculated from Eq. 6 with parameters 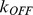 = 0.2, 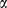 = 0.2, 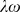 = 0.2, 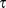 = 2s, 4s, 6s (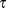 increased after every 10 images). The dotted curves show the predicted unobserved dynamics. (B) Fitting of 
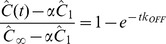
 to the normalized predicted data from A. The fitting yields 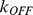  = 0.0846, much different from the actual value of 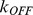 = 0.2.

Next the experiment was repeated in the same focal adhesion but under higher excitation laser intensities to induce more photobleaching of the cytoplasmic pool. The fluorescence recovery occurred with a marked decrease in the intensity at later times. The model again was able to describe the decrease in the intensities, and parameters could be estimated. Importantly, the 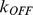 determined from the two different experiments matched very well (0.17 s^−1^ versus 0.18 s^−1^). Also, the 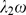 value determined from fitting in Figure 4E is close to that from fitting in Figure 4C (0.012 versus 0.009). This suggests that the model is able to estimate the kinetics of dissociation accurately despite the effects of photobleaching during image capture. When the data in Figure 4B was fit to the conventional model 
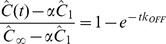
, the value of 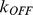 was found to be 0.3, a clear difference in the value obtained from the above model (Figure S1).

## Discussion

The analysis of FRAP experiments is an ongoing area of research. Among the many complicating factors [1], the effect of photobleaching by the image capture itself has not received much attention. In this paper, we propose an approach to account for this by explicitly including photobleaching into the modeling of the fluorescence recovery process. The method involves modeling the unobserved dynamics (which by definition are unaffected by photobleaching), and modeling the photobleaching during the period of observation. As the observation occurs at discrete time intervals (i.e. images collected at discrete time intervals), the photobleaching is modeled to occur at discrete time intervals superimposed on the unobserved dynamics that occur continuously.

A simple conclusion from the modeling is that the immobile fraction should not be calculated from the FRAP curve itself (as is common practice). Instead, the number of images should be minimized, preferably to only three images: one before bleach, one immediately after, and one when the recovery reaches a steady state (the characteristic time scale for the steady state can be established from separate FRAP experiments). As seen in Figure 4B, the value of 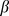 would be 0.48 if calculated from the FRAP experiment data (*) instead of 0.026 from an independent experiment (solid circles) with only three images collected. Another important concept is that the immobile fraction continues to be photobleached by the image capture process. Therefore, the FRAP curve is a combination of dynamics due to exchange of the mobile species, bleaching of the recovered portion due to the imaging process and the decay due to bleaching of the immobile fraction.

The main utility of this approach is when the bleaching during image capture significantly changes the FRAP dynamics. To test the extent of bleaching, the approach should be to first estimate the immobile fraction as described above. Then when the FRAP experiment is performed, the apparent immobile fraction from the FRAP experiment should be compared with the measured immobile fraction. A decrease from the actual immobile fraction indicates the extent to which photobleaching during image capture is relevant in the experiment. The effect of photobleaching may be unavoidable either due to the fact that the fluorophore may be particularly susceptible to bleaching or the intensity of the fluorophore in some cells may be lower than others requiring a higher excitation intensity leading to higher bleaching. In this situation, Eq. 9a and 9b should be used to fit the FRAP experiment. The parameters that are known in these equations are 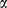, 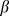 and 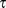 (measured or known directly from the experiment). The fitting should determine the values of 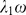, 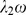 and 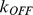. In situations where the diffusing cytoplasmic (or membranous) molecules can be tracked (such as in the example in Figure 4), it is useful to determine the value of 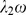 from fitting of the cytoplasmic pool, such that only two parameters need to be estimated.

An interesting prediction is that when the immobile fraction is present, the fluorescence in a FRAP experiment can reach a maximum and decay subsequently. The decay is due to the bleaching of the immobile fraction. Eventually, a steady state is reached when the bleaching due to image capture is compensated by recovery (unobserved dynamics). In the absence of the immobile fraction, the fluorescence reaches a steady state without reaching a peak. Thus the fact that the FRAP experiment reaches a steady state without any visible decay in the fluorescence does not imply lack of photobleaching during image capture; indeed it is possible to misinterpret lack of recovery in terms of an immobile fraction. If bleaching due to image capture is so severe that the free molecules (in the cytoplasm) are also bleached with each captured image, then there will be decay in the fluorescence and no steady state will be reached.

Sometimes researchers vary the time interval during FRAP experiments such that images are collected at a higher rate at the beginning of the recovery, and a smaller rate at later stages. We explored the prediction of Eq. 6 with parameters 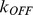 = 0.2, 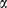 = 0.2, 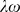 = 0.2, 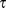 = 2s, 4s and 6s (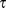
increased after every 10 images). At every increase of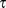, the steady state fluorescence recovery is predicted to increase (Figure 5A) leading to ‘bumps’ in the recovery process. The increase is due to a longer time interval between image captures that allows for more recovery in between successive image capture events (the bleaching due to image capture remains the same). If a conventional model 
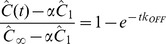
 is used to fit this model-generated data, the 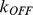
is estimated to be 0.085, which is much different from the real value 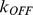 = 0.2 (Figure 5B).

This approach is applicable to more complicated situations. The method is to substitute Eq. 4 with the relevant model for the unobserved dynamics (for example, models that include equations for coupled transport and binding). The main concept is to replace the initial condition for the unobserved dynamics in between images with the bleaching-corrected concentration from the previous time interval. Thus, the approach is general and should work for any FRAP analysis.

If the values of the bleaching parameter 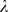 are calculated not from FRAP experiments, but from whole-cell imaging experiments with Eq. 3, then it is important to ensure identical imaging conditions for the corresponding FRAP experiments. This is because 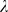 depends on experimental conditions; changing imaging conditions will change 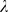, thereby invalidating the analysis for the FRAP experiment.

## Supporting Information

Figure S1
**Typical fitting for a GFP-VASP FRAP experiment.** The same FRAP experiment data as shown in Fig. 4B was fit to 
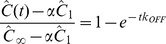
. The fitting yielded 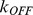  = 0.30.(TIF)

Supporting Information S1 **Model for the Bleaching of Free Protein in the Cytoplasm.**
(DOCX)
