# Supplementary figures and images for: FRAP Analysis: Accounting for Bleaching during Image Capture
Source: PLoS One. 2012 Aug 9;7(8):e42854. doi: 10.1371/journal.pone.0042854 (PMC3415426; doi:10.1371/journal.pone.0042854)

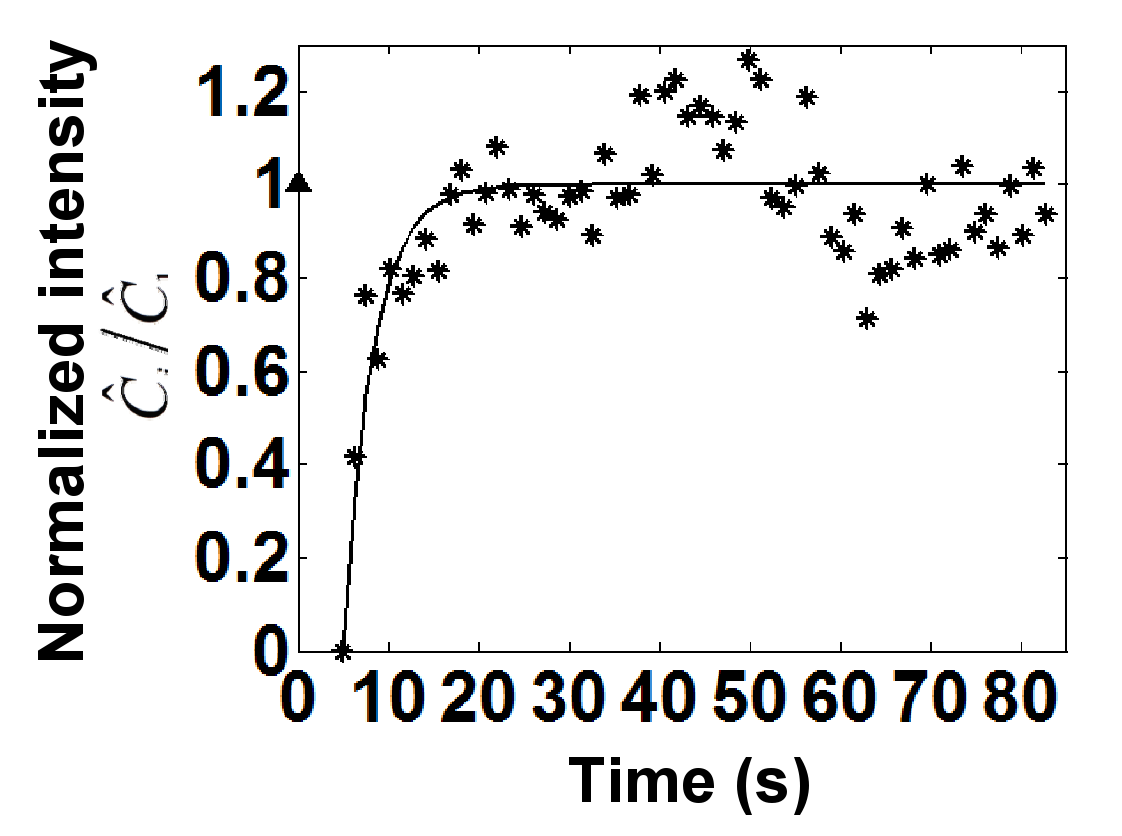

Supplement: Figure S1 — Typical fitting for a GFP-VASP FRAP experiment. The same FRAP experiment data as shown in Fig. 4B was fit to . The fitting yielded = 0.30. (TIF) [file pone.0042854.s001.tif]
