# Supplementary material for: FRAP Analysis: Accounting for Bleaching during Image Capture
Source: PLoS One. 2012 Aug 9;7(8):e42854. doi: 10.1371/journal.pone.0042854 (PMC3415426; doi:10.1371/journal.pone.0042854)
Supplement: Supporting Information S1 — Model for the Bleaching of Free Protein in the Cytoplasm. (DOCX) [file pone.0042854.s002.docx]

**Supporting Information**

***FRAP Analysis: Accounting for Bleaching during Image Capture***

Jun Wu^1^, Nandini Shekhar^1^, Pushkar P. Lele^2^, Tanmay P. Lele^1^*

^1^ Department of Chemical Engineering, University of Florida, Gainesville FL 32611

^2^ Department of Molecular and Cellular Biology, Harvard University, Cambridge MA 02138

*Address correspondence to: Tanmay P. Lele ([tlele@che.ufl.edu](mailto:tlele@che.ufl.edu))

Department of Chemical Engineering, Bldg 723,

University of Florida, Gainesville, FL 32611

Ph: 352-392-0317

**Figure Legends**

**Figure S1**. Typical fitting for a GFP-VASP FRAP experiment. The same FRAP experiment data as shown in Fig. 4B was fit to . The fitting yielded $k_{OFF}$ = 0.30.
